# Data on highway maintenance, rehabilitation, and mobility projects for integrated planning in Texas

**DOI:** 10.1016/j.dib.2019.104367

**Published:** 2019-08-05

**Authors:** Jojo France-Mensah, Chirag Kothari, William J. O'Brien, Nabeel Khwaja

**Affiliations:** a Department of Civil, Architectural and Environmental Engineering, The University of Texas at Austin, 301 E. Dean Keeton Street, ECJ 5.412, Austin, TX, 78712, USA; b Center for Transportation Research, The University of Texas at Austin, 1616 Guadalupe, Suite 4.202, Austin, TX, 78701, USA

**Keywords:** Highway projects, Maintenance and rehabilitation (M&R), Visualization, Cost allocation

## Abstract

State Highway Agencies (SHAs) have different functional groups that work towards improving the functional and physical performance of highway assets. These functional groups often propose multiple inter-related highway projects on the same network. However, the respective information systems of such functional groups lack interoperability capabilities between them. This data article is related to an earlier study by France-Mensah et al. (France-Mensah et al., 2017) that explored the integrated visualization of highway projects proposed by different functional groups working in the same highway agency. This dataset provides a spatially integrated set of maintenance and capital planning projects which is rarely available due to organizational silos which often exist in highway agencies. The data includes approximately 700 highway projects with over 16 attributes that includes spatial, temporal, cost, and description attributes. The highway projects are located in the Fort Worth District of the Texas Department of Transportation (TxDOT) which is responsible for a large network (approximately 9000 lane miles) of highway assets. The agency currently oversees around $4 billion in construction projects and spends around $120 million annually for asset preservation. An analysis of the fund allocations categorized by different project types for pavement and bridge assets is presented. The data presented can be used to compare competing approaches or policies for cross-asset allocation, spatial-temporal projects coordination, and safety planning in the infrastructure management domain.

**Table:** Specifications Table

Subject area	*Civil Engineering*
More specific subject area	*Transportation, Project Management*
Type of data	*Table, figure*
How data was acquired	*Aggregation and standardization of highway projects data extracted from the Design and Construction Information System (DCIS) of TxDOT and the Maintenance Management Information System (called COMPASS) of TxDOT*
Data format	*Raw, processed (cleaned, filtered, standardized), and analyzed*
Experimental factors	*Data of Highway projects that were planned for fiscal years 2016-2019 .Information included spatial, cost, and project description attributes.*
Experimental features	*Computation of a common spatial attributes of the projects to facilitate spatial conflict analysis of highway projects.*
Data source location	*Fort Worth, Texas, US*
Data accessibility	*The data is available with this article.*
Related research article	*The data is associated with an earlier publication*[1]*by the authors.*

**Table:** 

**Value of the data** • Highway projects cost data can be useful for studies on budget allocation in highway Maintenance and Rehabilitation (M&R) which require an actual dataset or context for comparison. • The breakdown of funds by project type can also be useful as a case comparison in studies on cross-asset allocation of highway funds for bridge and pavement assets. • The spatial information about M&R and mobility projects can be used in conjunction with other socio-economic factors as a case study to investigate equity implications of the proposed projects • Safety projects can also be employed in conjunction with open-source accidents data to perform a detailed analysis of spatial crash distributions and intervention (project) outcomes.

## 1 Data

The data presented is based on planned highway projects by the Fort Worth District of the Texas Department of Transportation (TxDOT). The district develops its 4-year Transportation improvement program after assessing the project needs and budget availability. However, the needs and budget availability are dynamic in nature and thus, the district updates its 4-year plan every four months. This dataset was received in the month of February 2016. Accordingly, some of the project details may have been modified since then.

The Fort Worth district oversees nearly $4 billion in construction and spends around $120 Million annually for M&R activities. The district area is around 7000 sq. miles and it caters to the demand of nearly 2.5 million residents [2]. For context, the highway network of the district is as large as the entire road network of the State of Hawaii. In terms of the area covered, the district is about the combined size of the States of Connecticut and Rhode Island. Major functional groups in this district are the Maintenance functional group and the Transportation, Planning, and Development (TP&D) functional group. The former group is responsible for routine and preventive maintenance projects like crack sealing, pothole repairs, seal coats, pavement leveling, and edge maintenance treatments. The Maintenance functional group documents such projects in their Maintenance Management Information System (referred to as COMPASS). Table 1 shows a breakdown of the funds allocated to different work categories for maintenance projects planned for the fiscal years 2016–2019. The major treatments include seal coats and preparatory works like pavement leveling, milling, base repair, and crack sealinsg.

**Table 1:** COMPASS project cost breakdown (2016–2019).

Treatment Category	No. of Projects	Total Cost	Percentage of Total Amount
Pavement Leveling	202	$ 51,377,029	58.21%
Milling	49	$ 1,002,819	1.14%
Base Repair	98	$ 12,614,685	14.29%
Spot Seal Coat	1	$ 29,762	0.03%
Full Width Seal Coat	66	$ 19,438,195	22.03%
Crack Sealing	17	$ 761,847	0.86%
Edge Maintenance	36	$ 868,353	0.98%
Adding or Widening Pavement	15	$ 2,161,859	2.45%
**Grand Total**	**484**	**$ 88,254,549**	**100%**

On the other hand, the TP&D functional group is responsible for some cost-intensive preventive maintenance projects, rehabilitation, and new construction (mobility) projects. Such projects are documented in the Design and Construction Information System. Table 2 shows a breakdown of funds allocation by project class which includes bridge and pavement asset projects. Bridge projects include bridge rehabilitation, widening, or replacement. Pavement asset projects include restoration, upgrades, widening, and new construction of freeway and non-freeway routes.

**Table 2:** DCIS project cost breakdown (2016–2020).

Project Class	Class Description	Number of Projects	Budget	Percent of Total Amount
SFT	Safety	47	$ 84,486,454	4.9%
OV	Overlay	36	$ 98,990,374	5.8%
SC	Seal coat	37	$ 11,804,973	0.7%
BR	Bridge replacement	7	$ 33,882,518	2.0%
WF	Widen freeway	8	$ 403,728,958	23.6%
RES	Restoration of existing road	14	$ 49,929,679	2.9%
INC	Interchange (new or reconstructed)	6	$ 629,351,958	36.8%
SP2	Super 2 highway design	4	$ 24,707,664	1.4%
RER	Rehabilitation of existing road	27	$ 98,860,438	5.8%
BWR	Bridge widening or rehabilitation	5	$ 18,077,840	1.1%
UGN	Grading, base, drainage, and pavement	2	$ 33,276,927	1.9%
MSC	Miscellaneous	13	$ 39,402,995	2.3%
NNF	New location non-freeway	2	$ 55,241,571	3.2%
WNF	Widen non-freeway	6	$ 129,172,668	7.5%
**Grand Total**		**214**	**$ 1,710,915,016**	

## 2 Experimental design, materials, and methods

**Fig. 1:**
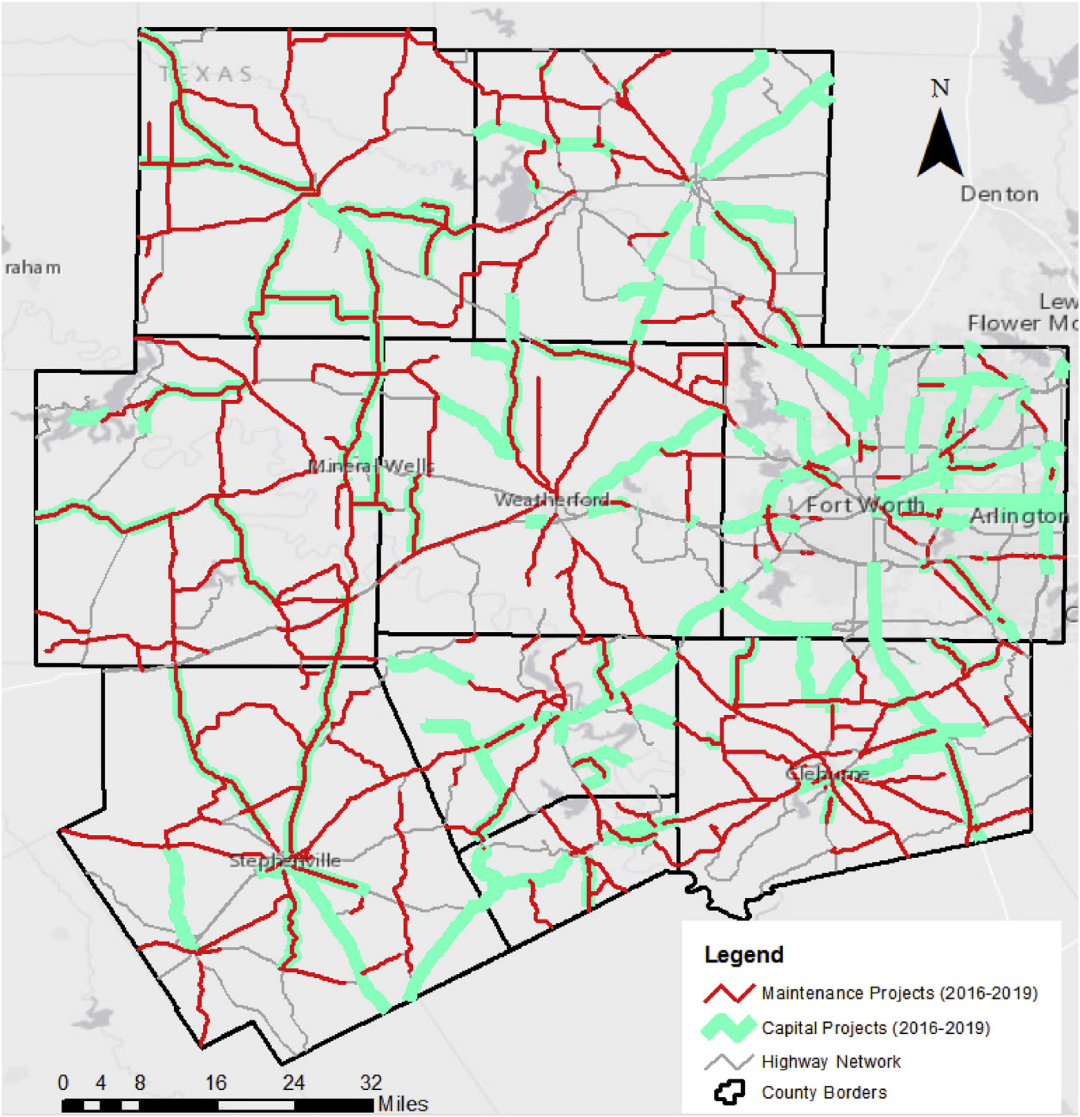
Map of maintenance and capital projects for fiscal years 2016–2019.

Raw projects data were extracted from the DCIS and COMPASS systems. However, the initially downloaded projects data were semantically heterogeneous in comparison to each other. Accordingly, a standardization process to ensure uniformity in input values and field names were conducted. Furthermore, while some fields contained the same information, the structure of the data was inconsistent. For example, Interstate 20 was represented as “IH 20” in one database but “IH0020” in another. The authors also standardized the activity descriptions of the COMPASS projects because there were semantically inconsistent descriptions of the same activity within the same field. From a geospatial standpoint, the raw spatial attributes had to also be converted to a GIS-compatible format for integrated visualization. Thus, the authors used another linear referencing system (LRS) that can be found in the Pavement Management Information System (PMIS) of TxDOT. The existing reference marker information from the projects were thus converted to the “Distance-from-Origin” LRS. To demonstrate the spatial connection between these initially disconnected set of projects, a GIS-based visualization of the projects is presented in Fig. 1. It is worth noting that projects without spatial information were excluded from the analysis presented. Also, routine maintenance projects related to vegetation control, littering, landscape enhancement, and traffic signals were excluded from the projects list. Thus, the totals presented do not necessarily represent the total budget for the agency but rather the relative expenditure of the district on different capital project types or maintenance activities.
